# Available and affordable complementary treatments for COVID‐19: From hypothesis to pilot studies and the need for implementation

**DOI:** 10.1002/clt2.12127

**Published:** 2022-03-12

**Authors:** Jean Bousquet, Tari Haahtela, Hubert Blain, Wienczyslawa Czarlewski, Torsten Zuberbier, Anna Bedbrook, Alvaro A. Cruz, Joao A. Fonseca, Ludger Klimek, Piotr Kuna, Boleslaw Samolinski, Arunas Valiulis, Antoine Lemaire, Josep M. Anto

**Affiliations:** ^1^ Institute of Allergology Charité – Universitätsmedizin Berlin Corporate Member of Freie Universität Berlin and Humboldt‐Universität zu Berlin Berlin Germany; ^2^ University Hospital Montpellier Montpellier France; ^3^ Allergology and Immunology Fraunhofer Institute for Translational Medicine and Pharmacology (ITMP) Berlin Germany; ^4^ Skin and Allergy Hospital Helsinki University Hospital University of Helsinki Helsinki Finland; ^5^ Department of Geriatrics Montpellier University Hospital, MUSE Montpellier France; ^6^ MASK‐air Montpellier France; ^7^ Fundaçao ProAR Federal University of Bahia and GARD/WHO Planning Group Salvador Bahia Brazil; ^8^ Department of Community Medicine Information and Health Decision Sciences (MEDCIDS) Faculty of Medicine, University of Porto Porto Portugal; ^9^ Patient‐Centred Innovation and Technologies Group (PaCeIT) Center for Health Technology and Services Research (CINTESIS) University of Porto Porto Portugal; ^10^ Medicina, EDucaçao, I&D e Avaliaçao Lda (MEDIDA) Porto Portugal; ^11^ Imunoalergologia CUF Porto Portugal; ^12^ Department of Otolaryngology, Head and Neck Surgery Wiesbaden Germany; ^13^ Center for Rhinology and Allergology Wiesbaden Germany; ^14^ Division of Internal Medicine, Asthma and Allergy Barlicki University Hospital Medical University of Lodz Lodz Poland; ^15^ Department of Prevention of Environmental Hazards, Allergology and Immunology Medical University of Warsaw Warsaw Poland; ^16^ Institute of Clinical Medicine and Institute of Health Sciences Medical Faculty of Vilnius University Vilnius Lithuania; ^17^ Centre Hospitalier Valenciennes France; ^18^ IMIM (Hospital del Mar Medical Research Institute) Barcelona Spain; ^19^ Universitat Pompeu Fabra (UPF) Barcelona Spain; ^20^ CIBER Epidemiología y Salud Pública (CIBERESP) Barcelona Spain; ^21^ ISGlobal Barcelona Institute for Global Health Barcelona Spain

**Keywords:** broccoli, COVID‐19, curcumin, Nrf2, TRP channel

## Abstract

Vaccination is a highly effective preventive measure against COVID‐19. However, complementary treatments are needed to better control the disease. Fermented vegetables and spices, agonists of the antioxidant transcription factor nuclear factor (erythroid‐derived 2)‐like 2 (Nrf2) and TRPA1/V1 channels (Transient Receptor Potential Ankyrin 1 and Vanillin 1), may help in the control of COVID‐19. Some preliminary clinical trials suggest that curcumin (spice) can prevent some of the COVID‐19 symptoms. Before any conclusion can be drawn and these treatments recommended for COVID‐19, the data warrant confirmation. In particular, the benefits of the foods need to be assessed in more patients, through research studies and large trials employing a double‐blind, placebo‐controlled design.

## 1 INTRODUCTION

Vaccination is a highly effective preventive measure against COVID‐19. However, it does not completely block the virus transmission and, although it reduces the severity of COVID‐19, it does not avoid all hospitalisations. Moreover, its effects decrease with time. Complementary treatments are therefore needed. Several expensive treatments are available, including monoclonal antibodies, but they are not readily available for most of the affected patients, particularly in developing countries. Drug repurposing could be cost‐effective, but, to date, no such treatment has been approved.

Several investigators have proposed that nutrients may play a role in preventing or controlling acute respiratory tract infections^1^ or COVID‐19.^2^ COVID‐19 may also be associated with a loss of biodiversity^3^ and gut microbiota changes.^3^

The COVID‐19 pandemic has opened fraught questions of equity on how the world has handled the pandemic. In addition to strategies providing universal access to vaccination, there is an urgent need for the global availability of simple and affordable treatments to complement vaccination.

## 2 THE UPDATED ANTI‐OXIDANT NRF2‐TRANSIENT RECEPTOR POTENTIAL CHANNEL HYPOTHESIS

It has been hypothesised that diet may partly explain large country variations in COVID‐19 death rates.^4^, ^5^ Some countries with low COVID‐19 death rates have a common habit of eating large quantities of fermented vegetables (such as cabbage) and various spices. The short duration of the Spring 2021 COVID‐19 outbreak in India due to the VOC‐δ (https://coronavirus.jhu.edu/map.html) may be related to diet.

**FIGURE 1:**
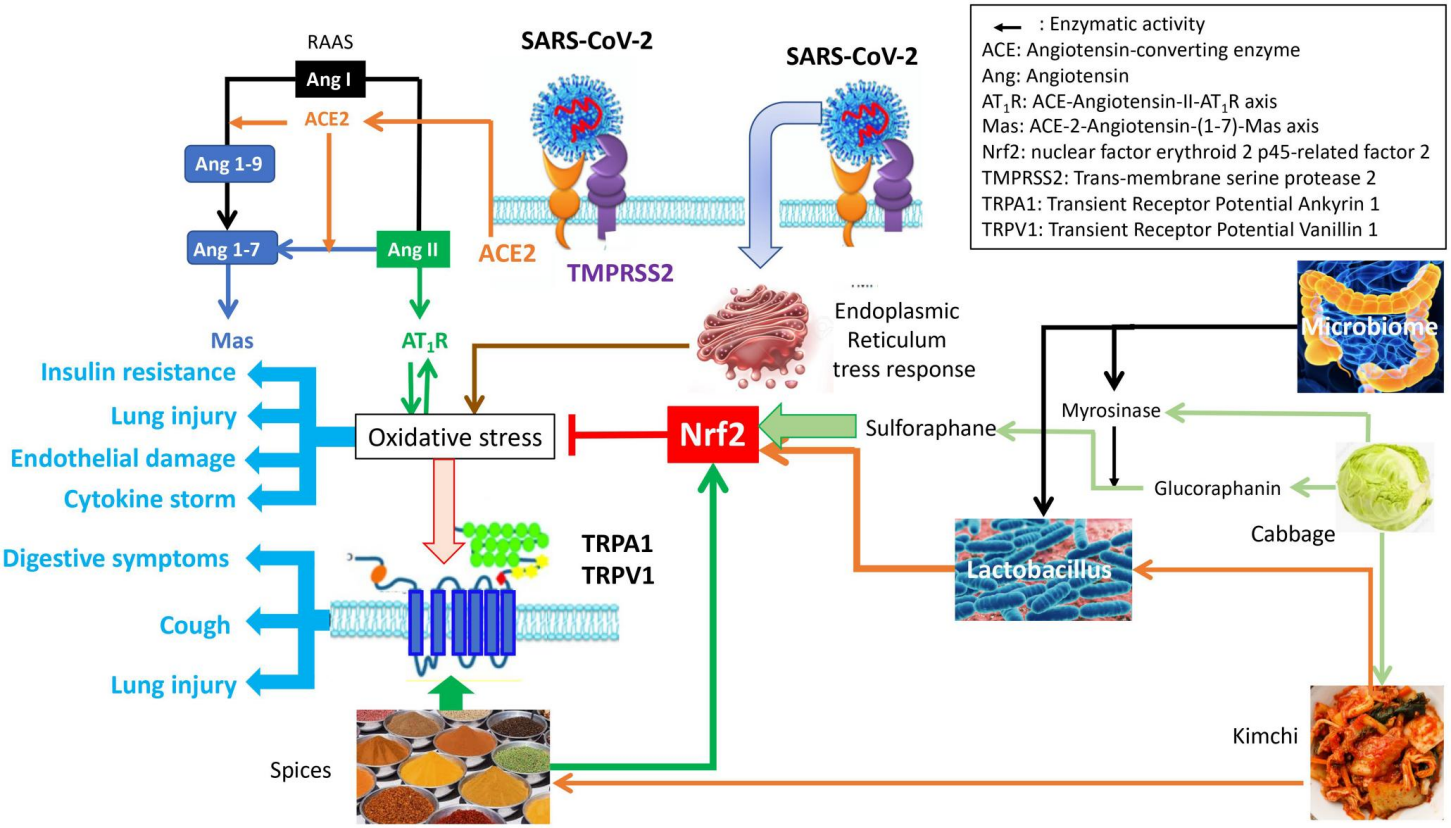
Putative mechanisms of Nrf2‐TRP agonists in the management of COVID‐19

One mechanism of COVID‐19 appears to be oxygen stress acting in synergy with transient receptor potential (TRP) channels.^6^ Fermented vegetables and spices are agonists of the antioxidant transcription factor nuclear factor (erythroid‐derived 2)‐like 2 (Nrf2), and spices are TRPA1/V1 (Transient Receptor Potential Ankyrin 1 and Vanillin 1) agonists.^7^ Mechanisms related to oxygen stress may explain many of the COVID‐19 symptoms as well as its severity.^8^, ^9^ The potential clinical relevance of Nrf2 and TPRA1/V1 therapies has been shown in a proof‐of‐concept paper including three clinical cases.^10^ Patients experienced a very rapid (minutes) improvement of some of the symptoms for a few hours. Cough and respiratory symptoms were improved better than loss of smell and taste. Induced‐cough challenges showed that broccoli capsules were effective within 10 min, whereas TRPA1/V1 agonists were effective within 1–2 min. Interestingly, paracetamol consistently increased the duration of action of curcumin and broccoli seeds, possibly through its TRPA1 activity. A synergy between Nrf2 and TRPA1/TRPV1 foods may explain the role of diet. Spicy foods are likely to desensitise TRP channels and act in synergy with exogenous anti‐oxidants that activate the Nrf2 pathway (Figure 1).^6^ The proof‐of‐concept studies suggest that some nutrients may be of interest in several stages of COVID‐19 and may partly cover an unmet medical need. Since the publication of the proof‐of‐concept, several small clinical studies have been reported.

## 3 CLINICAL STUDIES

### 3.1 Sulforaphane and Nrf2

The activation of Nrf2 has been proposed as a strategy against COVID‐19.^11^ ^,^ ^12^ Sulforaphane is the most potent natural activator of Nrf2. In vitro, sulforaphane inhibits the expression of IL‐6 and IL‐8 by a bronchial epithelial cell line exposed to the SARS‐CoV‐3 spike protein.^13^ Sulforaphane exhibits in vitro and in vivo antiviral activity against pandemic SARS‐CoV‐2 and seasonal HCoV‐OC43 coronaviruses.^14^ This study led to a patent filed by the Johns Hopkins University on a stabilised synthetic sulforaphane compound SFX‐01.^15^ A phase II study by Evgen Pharma was initiated using SFX‐01 to reduce acute respiratory distress syndrome (ARDS) associated with COVID‐19. Negative interim results on 133 patients with pneumonia led to the discontinuation of the study,^16^ suggesting that acting only on Nrf2 may be insufficient for the control of severe COVID‐19.

A trial in process (https://clinicaltrials.gov/ct2/show/NCT04421391) is assessing whether QuadraMune^®^, a food supplement containing sulforaphane, can prevent SARS‐CoV‐2 infection.

### 3.2 Curcumin

Curcumin has anti‐viral and immunomodulatory effects^17^ and inhibits SARS‐CoV‐2 in vitro.^18^, ^19^

A randomised clinical trial including 140 patients was carried out with curcumin (1050 mg) and piperine (5 mg) as an adjuvant therapy for COVID‐19.^20^ Patients with mild, moderate and severe symptoms who received a curcumin/piperine treatment (*N* = 70) showed inconstant early symptomatic recovery (fever, cough, sore throat, and breathlessness), less deterioration, fewer red flag signs, better ability to maintain oxygen saturation above 94% on room air, and better clinical outcomes compared to patients of the control group (*N* = 70).

A double‐blind, placebo‐controlled study was carried out in Iran on 40 COVID‐19 patients. 160 mg of Nano‐curcumin was administered daily for 14 days.^21^ To facilitate the application of curcumin and improve its stability and solubility, it was formulated with the aid of nanotechnology in Nano‐micelles that have interesting anti‐microbial properties.^22^ All of the COVID‐19 subjects in both groups received Betaferon for 5 days, Bromhexine every 8 h, and Atrovastatin daily. In a Per Protocol evaluation, fever, cough, dyspnoea (few patients) and chest radiographs were improved by Nano‐curcumin. After treatment with Nano‐curcumin, IL‐6 and IL‐1β gene expression and secretion were significantly decreased, but IL‐18 mRNA and TNF‐α were unchanged.

The efficacy of the oral Nano‐curcumin formulation in the management of mild to moderate outpatient COVID‐19 was investigated by a randomised triple‐blind, placebo‐controlled clinical trial.^23^ COVID‐19 patients from an outpatient setting who fulfilled the inclusion criteria were randomly allocated to the treatment (*N* = 30) group or to the placebo (*N* = 30) group. Patients of the treatment group received the oral nanocurcumin formulation (Sinacurcumin soft gel which contains 40 mg of curcuminoids as nanomicelles) and two soft gels twice a day after food for 2 weeks. All symptoms except sore throat resolved faster in the treatment group, and the difference was significant for chills, cough, as well as smell and taste disturbances. The CRP serum level was lower in the treatment group at the end of 2 weeks and the lymphocyte count was significantly higher. No substantial adverse reaction was reported in the treatment group.

An open, non‐randomised clinical trial assessed the efficacy of Nano‐curcumin in the management of 21 mild‐to‐moderate hospitalised COVID‐19 patients.^24^ By comparison with the control groups, most of the symptoms—including fever and chills, tachypnoea, myalgia and cough—resolved significantly faster in the curcumin group. Moreover, SaO_2_ was significantly higher in the treatment group after 2, 4, 7 and 14 days of follow‐up, as was the lymphocyte count after 7 and 14 days. The duration of supplemental O_2_ use and hospitalisation was also meaningfully shorter in the treatment group.

A trial in progress (https://clinicaltrials.gov/ct2/show/NCT05008003) is evaluating the combination of vitamin D, curcumin and quercetin on the early symptoms. The active comparator will be the standard of care.

## 4 SAFETY

### 4.1 Nrf2‐containing foods and supplements

Cruciferous vegetables such as broccoli are very healthy. No side effects (apart from some diarrhoea) have been reported using the regular dietary intake, except in certain patients with thyroid diseases.^25^ During the COVID‐19 pandemic, there were no reports of negative broccoli interaction.

It is considered that the daily intake of broccoli sprouts is around 30–50 g. Comparisons between sulforaphane and phenolic compounds of broccoli seeds and sprouts have been made. It appears that seeds contain 50%–70% of sprout compounds.^26^ In another study, broccoli seeds contained more glucoraphanin than sprouts (around 1.5‐fold).^27^ The glucoraphanin level in the broccoli seeds is largely determined by plant genotype, although the environment in which the plants are grown (e.g. location, year, drought, pollution and disease pressure) also plays a clear and significant role.^28^ Thus, it may be assumed that seeds contain between 0.5 and 1.5 g of bioactive components per gram of sprouts. A study published on ClinicalTrials.gov (ID: NCT03390855) showed that a daily consumption of 30 g of raw (not cooked), fresh broccoli sprouts during 10 weeks (70 days) was safe.^29^ The daily dose of broccoli seed capsules arbitrarily proposed in food supplements ranges from 300 to 1000 mg (less than 5% of the daily intake of broccoli sprouts).

Kimchi, a traditional Korean fermented food, usually contains cabbage and/or radish (Nrf2), garlic, red pepper, ginger and other spices (TRPA1/V1). Fermentation by lactic acid bacilli increases the health benefits of Kimchi (Nrf2).^30^ Koreans consume around 60 g of Kimchi per day^31^ and there have been no reports associating Kimchi with side effects in COVID‐19 patients.

In clinical studies, doses of up to 800 μmol daily of glucoraphanine have been reported without safety concerns.^29^, ^30^, ^31^

There have not been any reports of a negative interaction between broccoli or kimchi and COVID‐19.

Although there is no recommendation for pharmacologic doses issued in Europe or the United States for both broccoli and sulforaphane, broccoli may, at a very high dose, have some pharmacologic effects such as (i) the antagonism of aryl hydrocarbon receptors modulating the CYP1 (Cytochrome P450, family 1, subfamily A, polypeptide 1) family of cytochromes P450^32^ or (ii) a direct effect of CYP1.^33^ These effects must be studied carefully before performing trials at high doses. Moreover, in broccoli seeds, there are many other compounds that may have pharmacologic properties when ingested at high doses.^34^

### 4.2 Curcumin

Curcumin and black pepper are routinely used in the Asian cuisine at relatively high doses, and capsules are available in pharmacies or biofood shops. Curcumin has a long‐established safety record.^35^ According to the JECFA (Joint United Nations and World Health Organization Expert Committee on Food Additives) and EFSA (European Food Safety Authority) reports, the Allowable Daily Intake (ADI) value of curcumin is up to 3 mg/kg body weight based on the No‐Observed‐Adverse‐Effect Level (NOAEL).^35^, ^36^, ^37^ In the EFSA re‐evaluation of 2014,^37^ the dose of curcumin dietary food supplements, supplied in a solid form including capsules, should be 300 mg per day. Several trials on healthy subjects have supported the safety and efficacy of curcumin. Despite this well‐established safety, some negative side effects have been reported. Seven subjects receiving 500–12,000 mg in a dose–response study and followed for 72 h experienced diarrhoea, headache, rash and yellow stool.^38^ In another study, some subjects receiving 0.45–3.6 g/day of curcumin for one to 4 months reported nausea and diarrhoea as well as an increase in serum alkaline phosphatase and lactate dehydrogenase contents.^39^

Curcumin was found to have some effect on coagulation, in particular on platelets, but these effects were considered as beneficial in haemostasis, anticoagulation and fibrinolysis.^40^

A warning was issued for curcumin by the Anses (*Agence nationale de sécurité sanitaire, alimentation, environnement, travail*, France), the French agency for food safety, as it may impact SARS‐CoV‐2 infection.^41^ The Anses recommends the use of curcuma only in the frame of a clinical trial.

## 5 NEED TO SCALE UP THE RESEARCH ON NRF2‐TPRA1 CLINICAL THERAPIES

It is important to find simple, cheap and safe complementary treatments for COVID‐19. The results of these few clinical studies cannot yet be taken as formal evidence since the dietary patterns in India or Iran differ from those in Europe, and the sample size is small. Moreover, the proof‐of‐concept study in a very limited number of patients has led to the hypothesis that combined Nrf2‐TRPA1 foods may be beneficial for some COVID‐19 symptoms and that there is a synergy between Nrf2 and TRPA1 agonists. The study has suggested that there could be a very rapid onset of symptom relief.

Before any conclusion can be drawn and these treatments recommended for COVID‐19, the data warrant confirmation. In particular, the benefits of the foods need to be assessed in more patients, through research studies and large trials employing a double‐blind, placebo‐controlled design (Table 1).

**TABLE 1:** Research needs and clinical research priorities

**The mechanisms of action of the compounds tested** should be investigated using proper experimental studies.
**Higher priority clinical studies**
• **Impact on hospitalised patients:** A relatively small‐scale POC study could check whether the nutrients can improve cough and oxygen saturation (SaO_2_) within minutes in hospitalised patients, complementing the standard of care. Depending on the results, a DB‐PC‐RCT could assess the efficacy of these nutrients on the severity of symptoms, need for ICU and duration of hospitalisations (High priority).
• **Prevention and control of COVID‐19 in older people living in home care services**: Many people with severe outcomes and death are elderly and live in these settings.^46^ ^–^ ^48^ It would be of paramount importance to test whether these safe compounds can reduce the severity of COVID‐19 in infected people as well as the transmission to the other residents when there is an outbreak in a setting (high priority).
• **Reduction of symptoms in long COVID‐19**: Many patients suffer from long COVID‐19 symptoms with an impact on social life and work (high priority, relatively easy to be carried out).
**Lower priority clinical studies**
• **Prevention of severe COVID‐19 symptoms in COVID‐19 patients:** A large‐scale study is needed to assess whether patients who developed mild (and/or early) COVID‐19 may be prevented from developing severe COVID‐19 symptoms and/or hospitalisations (medium priority).
• **Prevention of COVID‐19 symptoms in asymptomatic SARS‐CoV‐2‐infected people:** A large‐scale study is needed to assess whether asymptomatic SARS‐CoV‐2‐infected people may be protected from developing COVID‐19 symptoms and deteriorating to severe COVID‐19 symptoms (low priority as this would be extremely difficult to do).
**Exploratory**
• **Prevention of SARS‐CoV‐2 infection**: Nrf2 was found to be involved in SARS‐CoV‐2 infection.^49^ It is possible that the long‐term use of large quantities of Nrf2 agonists such as curcuma or kimchi may prevent infection (exploratory).

In countries where large amounts of spices are eaten, the consumption of fermented vegetables is also high. This is the case for cassava in Africa or many fermented vegetables in Asia. Different types of fermented foods and spices are widely consumed in eastern Asian countries. Among them, kimchi is the most popular Korean traditional food. In such countries, it is possible that another form of TRP desensitisation, ‘tachyphylaxis’, may be important. This is the reduction or the disappearance of symptoms in response to repeated applications of agonists.^42^, ^43^, ^44^

SARS‐CoV‐2 variants of concern (VOCs) may have a different impact on cells of the respiratory tract, and the SARS‐CoV‐2 B.1.1.529 *Omicron* variant may cause attenuated lung disease.^45^ The hypothesis should therefore be tested with all VOCs.

## 6 CONCLUSION

Without any doubt, in all infectious diseases, vaccination is the most important prophylactic treatment. However, in the case of infection, other treatment options are needed. Complementary treatments will never replace vaccination but can help to better control COVID‐19 and might even reduce SARS‐CoV‐2 infection. Complementary treatments with plant‐derived nutrients can help to better control COVID‐19 and might even reduce SARS‐CoV‐2 infection. These treatments would certainly not replace any appropriate treatment but do have the potential to reduce morbidity and shorten time of sick leave. These nutrients have the advantage of being available and affordable globally. Curcumin as a TRPA1/V1 agonist and Nrf2 agonists may represent prototypes for other nutrients. They certainly merit further investigation in various aspects of COVID‐19.

## CONFLICT OF INTEREST

The authors declare no conflict of interest.

## AUTHOR CONTRIBUTIONS

Jean Bousquet: Writing – original draft; Writing – review & editing. Tari Haahtela: Writing – review & editing; Hubert Blain: Writing – review & editing. Wienczylslawa Czarlewski: Writing – review & editing; Torsten Zuberbier: Writing – review & editing. Anna Bedbrook: Writing a– review & editing. Alvaro Cruz: Writing – review & editing). Joao Fonseca: Writing – review & editing. Ludger Klimek: Writing – review & editing. Piotr Kuna: Writing – review & editing. Boleslaw Samolinski: Writing – review & editing. Arunas Valiulis: Writing – review & editing. Antoine Lemaire: Writing a – review & editing. Josep M. Anto: Writing – review & editing.
